# Acute effects of supramaximal loaded back squat activation on countermovement jump performance, muscle mechanical properties, and skin surface temperature in powerlifters

**DOI:** 10.1002/ejsc.12245

**Published:** 2024-12-31

**Authors:** Dawid Perenc, Petr Stastny, Robert Urbański, Michał Krzysztofik

**Affiliations:** ^1^ Nutrition and Sports Performance Research Group The Jerzy Kukuczka Academy of Physical Education in Katowice Katowice Poland; ^2^ Department of Sport Games Faculty of Physical Education and Sport Charles University in Prague Prague Czech Republic; ^3^ Institute of Sport Sciences, The Jerzy Kukuczka Academy of Physical Education in Katowice Katowice Poland; ^4^ Department of Biomechanics and Sports Engineering Gdansk University of Physical Education and Sport Gdańsk Poland

**Keywords:** accentuated eccentric loading, muscle strength, post‐activation performance enhancement, post‐activation potentiation, power output, weight releasers

## Abstract

This study aimed to investigate the effects of performing either eccentric‐only (ECC) or eccentric‐concentric (ECC‐CON) back squats (BS) with a supramaximal load on countermovement jump (CMJ) performance. Changes in front thigh skin surface temperature and mechanical properties (oscillation frequency and stiffness) of the vastus lateralis were also examined. Fourteen male powerlifters participated in this study (age: 22.5 ± 2.3 years, body weight: 84.2 ± 11.1 kg, height: 178 ± 7 cm, training experience: 5.4 ± 1.6 years, BS one‐repetition maximum [1RM]: 177 ± 22.8 kg). The experimental sessions included 2 sets of 2 BS at 110% 1RM of either ECC‐CON (load distributed by half on the barbell [55%] and on weight releasers [55%]) or ECC (only eccentric phase of BS) and CTRL with no CA applied. CMJ performance, mechanical properties, and skin surface temperature were measured before and at the third, sixth, ninth, and 12^th^ min. After each protocol, only the ECC‐CON condition led to a significant increase in CMJ height after individual optimal rest time compared to pre‐CA (38.1 ± 5.2 vs. 39.8 ± 5.0 cm; *p* = 0.003; effect size [ES] = 0.32; *Δ* = 4.9 ± 5.0%) with a significant rise in skin surface temperature (32.98 ± 1.24 vs. 33.69 ± 0.96°C; *p* = 0.006; ES = 0.62; *Δ* = 2.2 ± 2.6%) and no significant changes in mechanical properties of the vastus lateralis. The ECC‐CON condition led to a significant acute improvement in CMJ height and an increase in front thigh skin surface temperature among powerlifters. The ECC‐CON supramaximal lower limb PAPE protocol should be effectively used among males representing high levels of lower limb muscle strength (>2 × body mass).

## Highlights

- Supramaximal eccentric back squat activation protocol can be effectively used to acutely enhance countermovement jump height among males representing very high levels of lower limb muscle strength (>2 × body mass).
- Large variability in post‐activation performance enhancement responses observed across athletes suggests that it is crucial to individually assess the effectiveness of the chosen conditioning activity with assessments of the optimal rest interval in order to establish whether athletes respond positively and could experience athletic performance enhancement.

## 1 INTRODUCTION

A significant and acute improvement in athletic performance can be achieved through the post‐activation performance enhancement (PAPE) effect (Blazevich et al., 2019). In practice, this phenomenon is used in complex training by combining pairs of exercises (strength‐power potentiating complexes). For example, a high‐intensity conditioning activity (CA) (∼80% of one‐repetition maximum [1RM]) and then, after an appropriate rest interval (typically 5–7 min), performing an explosive task with a similar movement pattern, that is, a vertical jump (Wilson et al., 2013).

A wide range of CAs has been examined to date including plyometric, ballistic, and resistance exercises among various athletes (Bartolomei et al., 2023; Conrado de Freitas et al., 2021; Guerra et al., 2022; Krzysztofik et al., 2022). Typically, resistance exercises involve coupled eccentric‐concentric contractions with identical loads, limiting the load to the capacity of the concentric contraction. To achieve the desired level of intensity during the eccentric phase, supramaximally loaded eccentric‐only contractions can be employed. However, these settings do not permit loading the concentric phase. Specialized equipment, such as weight releasers (WR), can be used to impart varying weights throughout different loads, effectively targeting both the eccentric and concentric phases. WR detaches automatically from the barbell upon contact with the ground. This allows the use of a supramaximal load that detaches upon contact with the ground, leaving a submaximal load for the concentric phase, thus appropriately loading the concentric phase. This method also enables the utilization of the stretch‐shortening cycle, which in most exercises involves both eccentric and concentric muscle actions performed in succession (Bobbert et al., 1996).

There is a notable interest in incorporating eccentric contractions into CAs because of their unique features, such as their capacity to use loads significantly surpassing those possible to apply during concentric and isometric contractions. In addition, supramaximal eccentric contractions are thought to selectively recruit higher‐order motor units to a greater extent than concentric contractions (Desmedt et al., 1977). They also have the potential to reduce inhibitory reflexes and increase rate coding to a greater degree compared to submaximal loads (Douglas et al., 2017). In addition, the energy demands for eccentric contractions are roughly four times less than those for concentric contractions (Hoppeler, 2016). Therefore, it is reasonable to consider that there could be a higher level of acute activation for a given level of fatigue while also improving the balance between activation and fatigue, plausibly leading to the PAPE effect (Gossen et al., 2000). Consequently, activation due to eccentric contractions may induce a relatively higher PAPE effect compared to other types of contractions due to increased activation and either lower or similar levels of fatigue. Studies on the use of eccentric‐only CAs showed an acute increase in power output during athletic movements as long as the load exceeds 100% of the concentric 1RM (Krzysztofik et al., 2020, 2022; Ong et al., 2016). On the other hand, eccentric contractions, performed by unfamiliarized participants against supramaximal loads may lead to muscle micro lesions and result in performance decline (Hedayatpour et al., 2015). Therefore, the use of supramaximal eccentric exercise as a CA may vary depending on an athlete's strength level and experience (Suchomel et al., 2019a, 2019b). For example, Krzysztofik et al. (2020) found improved bench press throw performance after supramaximal loaded bench presses in strong males (>1.5 × body mass). However, there is a lack of similar studies on the lower limb PAPE protocol in very strong groups of participants (i.e., >2 × body mass in back squat [BS]) along with an attempt to determine the underlying mechanisms of performance enhancements.

Mechanisms of the PAPE effect are thought to be explained by increases in muscle temperature, muscle water content, and potentially increased phosphorylation of the myosin regulatory light chain due to intense muscle contractions during CA, resulting in increased sensitivity of actin and myosin fibers to Ca^2^+ calcium ions (Blazevich et al., 2019). Given that confusion exists with respect to the physiological mechanisms, the PAPE effect has also attracted interest in the scientific world. The latest studies focused on assessments of changes in muscle stiffness and tone (oscillation frequency) (Krzysztofik, Spieszny, et al., 2023; Krzysztofik, Wilk, Pisz, Kolinger, Bichowska, et al., 2023; Krzysztofik, Wilk, Pisz, Kolinger, Tsoukos, et al., 2023) which might be attributed to intramuscular fluid pressure changes (Korhonen et al., 2005). Interestingly, these studies collectively indicate that performance improvements were noted when muscle stiffness and tone remained the same or decreased (Krzysztofik, Spieszny, et al., 2023; Krzysztofik, Wilk, Pisz, Kolinger, Bichowska, et al., 2023; Krzysztofik, Wilk, Pisz, Kolinger, Tsoukos, et al., 2023), and the increase in these variables was associated with a decrease in performance (Klich et al., 2020; Trybulski et al., 2022). On the other hand, in terms of skin surface temperature, there are even fewer studies available with inconsistent findings (Baena‐Raya et al., 2023; Krzysztofik, Wilk, Pisz, Kolinger, Tsoukos, et al., 2023). A recent study indicates that an increase in CMJ height was accompanied by an increase in front thigh skin surface temperature following a CA protocol involving 3 sets of 3 repetitions at 85% 1RM and 2 sets at 60% 1RM until a mean velocity loss of 10% (Krzysztofik, Wilk, Pisz, Kolinger, Tsoukos, et al., 2023). Conversely, the same study observed an improvement in CMJ height with a decrease in front thigh skin surface temperature following a single set of BS at 60% 1RM until a mean velocity loss of 10% (Krzysztofik, Wilk, Pisz, Kolinger, Tsoukos, et al., 2023). In contrast, Baena‐Raya et al. (2023) noted an immediate decrease in skin surface temperature (mean temperature measured at eight anatomical points across the body) along with a decrease in CMJ height following varied low‐volume BS protocols at 60% 1RM (2 sets of 6 repetitions, 2 sets of 6 repetitions with 30‐s interrepetition rest every 2 repetitions, single set of 12 repetitions with 36‐s interrepetition rest every repetition). On the other hand, there is no such evidence after supramaximal loaded CAs. Taking into account that a very low volume of supramaximal CAs was sufficient to elicit the PAPE effect in previous studies (Krzysztofik et al., 2020, 2022), it seems that they will not contribute to significant changes in skin surface temperature and intramuscular fluid pressure. Therefore, if the PAPE effect will be noted, then perhaps other mechanisms will underpin the changes in performance.

Considering the small number of studies on the PAPE effect examining the supramaximal loaded CAs, this study aimed to determine the potential mechanisms underlying the improvement of physical fitness among participants representing very high muscle strength levels. Specifically, the objective was to compare the effectiveness of supramaximal loaded eccentric‐only and eccentric‐concentric BS in powerlifters able to squat above two times their body mass. In addition, the stiffness and muscle tonus of the vastus lateralis, as well as the skin surface temperature, were measured. It was hypothesized that both CAs would contribute to the improvement of subsequent CMJ, with no changes in skin surface temperature and a decrease in vastus lateralis stiffness and tonus.

## 2 MATERIALS AND METHODS

### 2.1 Experimental approach to the problem

A single‐blind experimental design was used in the study. Participants attended a familiarization session and three experimental sessions in a randomized order. Randomization was performed using an online generator. The familiarization session consisted of determining the level of maximal strength in the BS and familiarizing the participants with the procedure of the upcoming experimental sessions. The experimental sessions were performed at an interval of 1 week, during which each participant performed i) ECC‐CON—2 sets of 2 repetitions of the BS with an external load of 110% 1RM distributed 55% on the barbell and 55% on the WR; ii) ECC—2 sets of 2 repetitions of the eccentric phase only of the BS with a load of 110% 1RM; and iii) CTRL—without performing CA. To assess the acute effect of those CAs, the biomechanical properties of the vastus lateralis, surface skin temperature of the front thigh, and CMJ performance were measured 5 min before and at the third, sixth, 9^th^, and 12^th^ minute after each CA.

### 2.2 Participants

Fourteen male powerlifters (age: 22.5 ± 2.3 years, body mass: 84.2 ± 11.1 kg, height: 178 ± 7 cm, training experience: 5.4 ± 1.6 years, BS 1RM: 2.10 ± 0.12 kg/body mass, bench press 1RM: 1.77 ± 0.1 kg/body mass, deadlift 1RM: 2.27 ± 0.17 kg/body mass) participated in this experiment. The following criteria were used to select participants for the study: i) muscle strength level in the BS > 2 × body mass, ii) a minimum of 4 years of experience in resistance training, and iii) regular participation in resistance training, including supramaximal BS and use of WR. Participants were instructed to maintain their usual dietary and sleeping habits and to abstain from stimulants and alcoholic beverages for the duration of the study. They were also instructed not to do any additional resistance exercises in the 48 h before the study to avoid fatigue. Participants were free to withdraw at any time. They were fully informed of the potential risks and benefits of the study before giving written informed consent; however, they were not told about the expected outcomes. The research protocol was approved by XXXX and conducted in accordance with the Declaration of Helsinki from 2013.

### 2.3 Countermovement jump assessment

The CMJ assessment was conducted on force plates (Force Decks, Vald Performance, Australia), a validated and reliable device for measuring vertical jump performance (Heishman et al., 2020). The following variables were recorded: i) jump height based on take‐off velocity [cm], ii) relative maximum power [W/kg], iii) time to take‐off [ms], and iv) countermovement depth [cm]. Participants performed 2 CMJ trials at each time point, 5 min before and at the third, sixth, ninth, and 12^th^ min after CA. To obtain body mass, participants assumed a 3‐s quiet standing period with the trunk upright, knees fully extended, feet shoulder‐width apart, and hands resting on the hips (throughout the attempt). Then, they were instructed to execute a downward movement to a self‐depth, followed by a powerful upward movement to reach the maximum jump height. After each jump, the participant returned to the starting position and the procedure was repeated. The highest CMJ height was selected for further analysis. The jumping height was determined by using the following equation:

$$Jump\;height=\frac{1}{2}\cdot \left(\frac{{TOV}^{2}}{g}\right)$$

where TOV—vertical velocity of the center of mass at take‐off; and $g=9.81\;m\cdot \sec^{-2}$

### 2.4 Measurement of skin surface temperature

The skin surface temperature of the front thigh was measured using a thermal imaging camera (Flir E54, FLIR SYSTEM, Wilsonville, OR, USA) with a resolution of 320 × 240 pixels and a sensitivity of 0.05 K. The camera was calibrated by a black body; the emissivity was set at the range of 0.97–0.98 according to the Glamorgan protocol (Ammer, 2008) and according to a checklist for standardizing thermographic imaging in sports and exercise medicine (Moreira et al., 2017).

During measurements, the participants stood perpendicular at a distance of 1.5 m from the camera in front of a white uniform background. There was a constant room temperature (21° C), humidity (60%), intensity of light, and no direct ventilation in the test room. The camera was mounted on a tripod approximately at a height of participants' thighs to prevent image movement during the measurement. The mean temperature in the region of interest, corresponding to the area of the vastus lateralis, was determined using a dedicated software (Flir Thermal Studio, Flir System, Wilsonville, OR, USA). Thermal images were taken immediately before each CMJ attempt.

### 2.5 Measurement of viscoelastic muscle properties

A hand‐held myotonometer (MyotonPRO, Myoton AS, Tallinn, Estonia) was used for noninvasive measures of the biomechanical properties of the dominant limb vastus lateralis (muscle tone [oscillation frequency] and stiffness). The measurement was performed at 50% of the straight‐line distance between the greater trochanter and fibulae capitulum (Bizzini et al., 2003) and marked with a non‐washable marker. Participants were instructed to mark this point in order to accurately reproduce the measurement point in the upcoming session. The accelerometer of the Myoton device was set at 3200 Hz, and the average of three consecutive measurements (0.4 N for 15 ms) was used for further analysis.

### 2.6 Familiarization session and 1RM back squat test

The familiarization session took place 1 week prior to the first experimental session and was designed to determine 1RM in the BS. All participants performed a standardized warm‐up prior to the experiment, which consisted of 5‐min cycling on an ergometer with an upper‐body engagement component (Keiser M3i Total Body Trainer, Keiser Corporation, Fresno, California) using a resistance that allows to achieve a power output of 100 W and a cadence of 70–80 rpm. This was followed by a specific lower body warm‐up consisting of 2 circuits of 10 bodyweight squats, 10 hip thrusts, 5 reverse lunges, 5 side lunges, and 2 submaximal vertical jumps. There were no rest intervals between exercises, but a 2‐min rest interval was allowed between circuits. Following the warm‐up, participants performed 10, 6, 4, and 3 repetitions of BS starting with a load of 20 kg and gradually increasing to an estimated value of 80% of 1RM (self‐declared). The initial test load was 90% of the estimated 1RM and was increased between 2.5 and 10 kg on each subsequent attempt depending on the participant and repeated until the participant was unable to complete the exercise with the correct technique. The 1RM was determined within 5 sets of 1 repetition, and a 5‐min rest interval between sets was adopted. All trials were performed with a controlled eccentric phase duration of 3 s (to ensure it was consistent with the eccentric phase during the CA in the main session), while for the concentric phase, participants were instructed to perform it as quickly as possible. Two certified strength and conditioning coaches were present during the 1RM test procedure.

Participants started the exercise from a standing position with knees and hips fully extended, shoulder‐width apart, and both feet placed flat on the ground in parallel or with a maximum external rotation of approximately 15°. The barbell was placed on the back at the shoulder blades, while the distance and positioning of the feet were individually adjusted and then repeated during each subsequent experimental session. From an upright position, participants performed the eccentric phase of the BS until the hip was lowered below the knee line and then performed the concentric phase without stopping. Except for powerlifters' shoes, any other training equipment such as a powerlifting belt, neoprene knee wraps, or other training equipment was not permitted. The exercise was conducted in accordance with the rules of the International Powerlifting Federation (IPF).

### 2.7 Conditioning activities

After the same warm‐up as in the familiarization session, participants performed 3 additional BS sets at 50%, 70%, and 90% of 1RM (8, 4, and 1 repetition, respectively), with 3‐min rest intervals. Afterward, participants performed either CA consisting of i) ECC‐CON condition of 2 sets of 2 repetitions of BS at 110% 1RM distributed by half (55/55%) on the barbell and on the WR (according to the recommendations of Merrigan et al. (2022) for the use of AEL to improve concentric velocity and power output in athletes with a relative strength >2.0 in the back squat); ii) ECC condition of 2 sets of 2 repetitions of the eccentric‐only phase of BS at 110% 1RM; or iii) CTRL condition without CA performed but treadmill walking with equivalent time. A 3‐min rest interval between sets was allowed. All measurements during the CTRL condition were taken at the same time points as in the other conditions. During the ECC‐CON, the participant performed the eccentric phase of the BS, and immediately after the release of WR, the concentric phase was performed. At the end of the repetition, two experienced strength and conditioning coaches reattached the WR to the barbell and the participant performed the next repetition in the same manner. During the ECC, the participant performed only the eccentric phase of the BS after which the spotters lifted the barbell onto the racks and the participant proceeded to the next repetition. During the ECC and ECC‐CON conditions, participants were instructed to complete the eccentric phase of the exercise in 3 s. In case of the ECC‐CON condition, the concentric phase was performed as quickly as possible.

## 3 STATISTICAL ANALYSES

All statistical analyses were performed using the SPSS software for MacOS (version 25.0; SPSS, Inc., Chicago, IL, USA) and presented as means with standard deviations (±SD). Furthermore, 95% confidence intervals (95%CI) were calculated for mean values and mean differences (*δ*). Moreover, relative differences (in percentages) between baseline and post‐CA values were presented. Statistical significance was set at *p* < 0.05. The Shapiro–Wilk and Mauchly's tests were applied to assess the normality and sphericity of variance in the sample data.

Additionally, based on the baseline values of particular variables recorded during experimental sessions, the relative (two‐way mixed effects, absolute agreement, and intraclass correlation coefficient [ICC] of a single rater) and absolute reliability (coefficient of variation) were calculated. The interpretation thresholds for intraclass correlation coefficient (ICC) results were interpreted based on the reported 95% CI of the estimated ICC as follows: <0.5 ″poor", 0.5–0.75 ″moderate", <0.76–0.9 ″good", and >0.90 as “excellent" (Koo et al., 2016). As for the coefficient of variation “acceptable", the threshold was set at ≤5%.

Furthermore, a minimal detectable change (MDC) calculated using the following formula (9)

$$MDC=SEM\times 1.96\times \sqrt{2}$$

was employed to define responders and nonresponders to the CA. A participant was considered i) a responder if the increase in CMJ height exceeded the MDC value, ii) a nonresponder if the change in CMJ height fell within the MDC value, and iii) a negative responder if the decrease in CMJ height surpassed the MDC. A chi‐square test was conducted to determine if there were significant differences among responders, nonresponders, and negative responders to the applied CA.

Finally, a two‐way analysis of variance (ANOVA) (3 [conditions] × 5 [time]) was employed to ascertain the effect of CA on the vastus lateralis muscle tone and stiffness and skin surface temperature of the front thigh, as well as the height, time to take‐off, countermovement depth, and relative power output during CMJ. Due to the high interindividual variability in potentiation responses and the individualized recovery time approach (Boullosa et al., 2020), an additional two‐way ANOVA (3 [conditions] × 2 [time]) was used to assess the impact of CA on the aforementioned variables, considering only the individual peak PAPE response. The eta squared (η^2^) value was used to evaluate the effect size for the interactions and main effects. In case of significant main effects or interactions, post hoc tests with Bonferroni corrections were employed for pairwise comparisons. The magnitude of mean differences was expressed using common language effect size (CLES).

## 4 RESULTS

The sample size was determined using G*Power software version 3.1.9.2 (Dusseldorf, Germany) with the following statistical test parameters: “repeated measures ANOVA within, factors" (one group of participants, three conditions, and five measurements), statistical power set at 0.8, and significance level of 0.05, based on the changes observed for CMJ height (40.77 ± 4.66 vs. 42.58. ± 4.74 cm) following PAPE protocols in the study by Krzysztofik, Wilk, Pisz, Kolinger, Tsoukos, et al. (2023). The analysis revealed that a minimum sample size of 10 participants was required for this study.

The MDC for CMJ height was 0.83 cm. The relative reliability assessed based on the lower bound of the ICC and coefficient of variation values were “good” (ICC = 0.93, 95%CI: 0.83–0.97) and 4.6% (95%CI: 3.5–5.8) for jump height, “moderate” (ICC = 0.89, 95%CI: 0.73–0.96) and 3.5% (95%CI: 2.4%–4.8%) for relative peak power output, “good” (ICC = 0.89 with 95%CI: 0.77–0.96) and 4.9% (95%CI: 3.6–6.4) for time to take‐off, “moderate” (ICC = 0.82 with 95%CI: 0.73–0.89) and 5.1% (95%CI: 3.9–6.4) for countermovement depth, “moderate” (ICC = 0.88 with 95%CI: 0.71–0.96) and 1.4% (95%CI: 0.9‐0.19) for skin surface temperature, “moderate” (ICC = 0.89 with 95%CI: 0.74–0.96) and 4.4% (95%CI: 2.5–6.3) for oscillation frequency, and “good” (ICC = 0.95 with 95%CI: 0.89–0.98) and 4.2% (95%CI: 2.6–5.9) for stiffness.

The chi‐square test indicated that the differences among responders, nonresponders, and negative responders to the applied CA were statistically significant (*χ* ^2^ = 9.85, *p* = 0.043). There were 9 responders, 4 nonresponders, and 1 negative responder in the ECC‐CON condition, while in the ECC condition, there were 4 responders, 5 nonresponders, and 5 negative responders. During the CTRL condition, 4 participants were considered as responders, 9 as nonresponders, and 1 as a negative responder.

### 4.1 Countermovement jump performance

**FIGURE 1:**
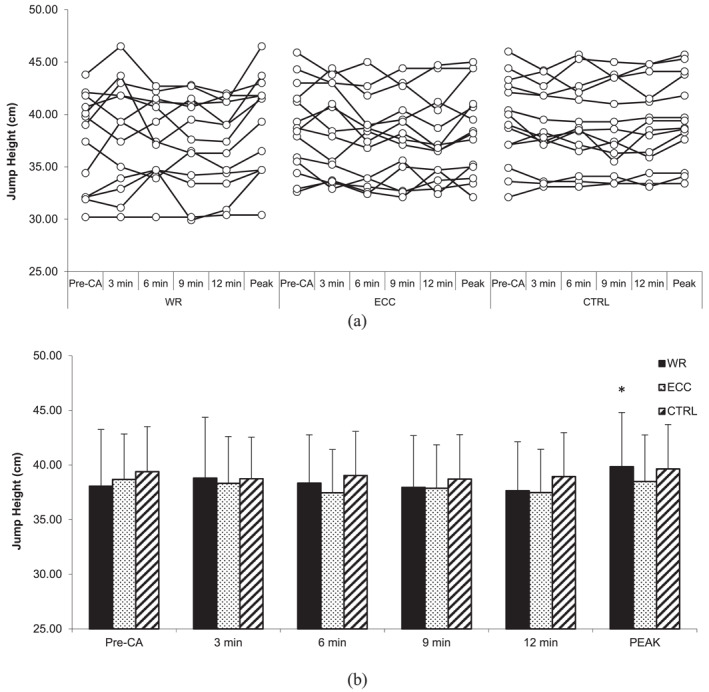
Comparison of pre‐CA and post‐CA countermovement jump height. Results are presented as individual responses (A) and mean ± *SD* (B); CA—conditioning activity; PEAK—optimal rest time interval; WR—eccentric‐concentric condition; ECC—eccentric‐only condition; CTRL—control condition. * significantly higher than pre‐CA protocol.

**FIGURE 2:**
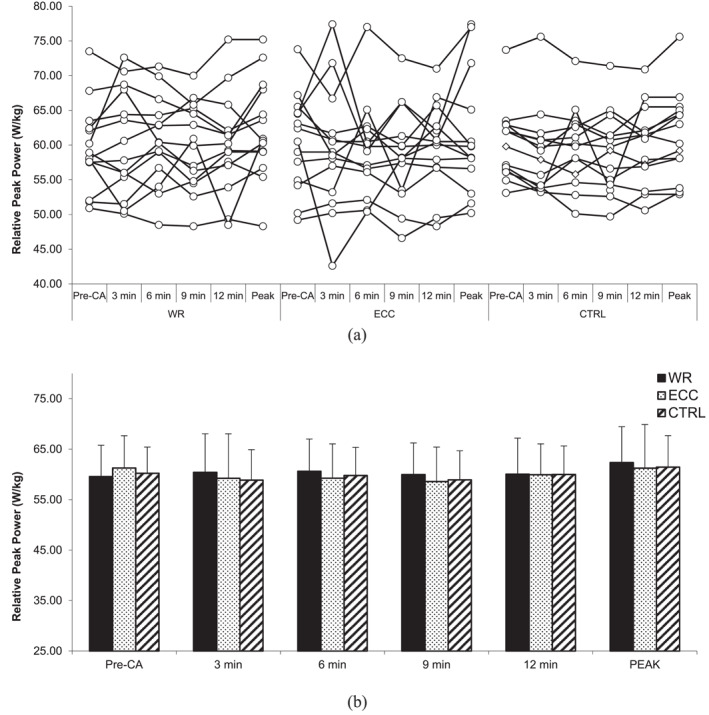
Comparison of pre‐CA and post‐CA countermovement jump relative peak power output. Results are presented as individual responses (A) and mean ± *SD* (B); CA—conditioning activity; PEAK—optimal rest time interval; WR—eccentric‐concentric condition; ECC—eccentric‐only condition; CTRL—control condition.

**FIGURE 3:**
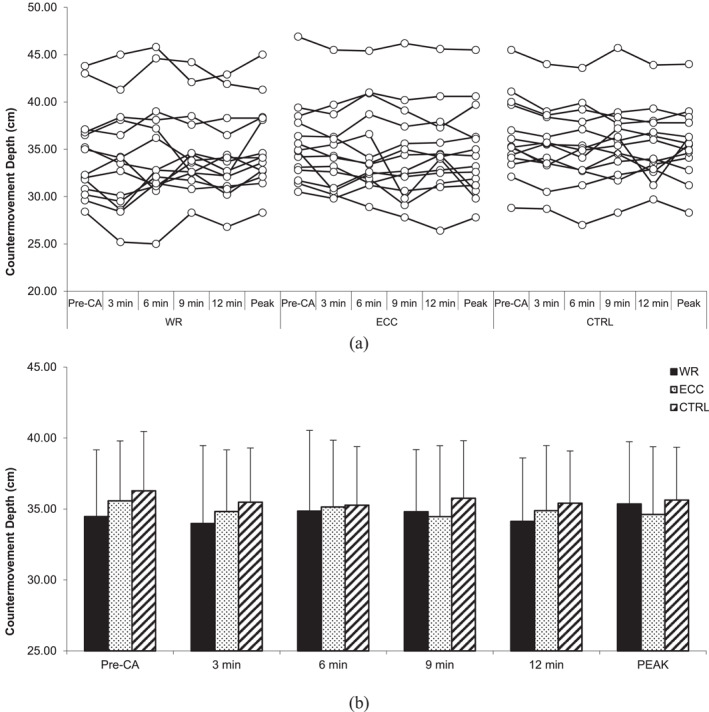
Comparison of pre‐CA and post‐CA countermovement jump depth. Results are presented as individual responses (A) and mean ± *SD* (B); CA—conditioning activity; PEAK—optimal rest time interval; WR—eccentric‐concentric condition; ECC—eccentric‐only condition; CTRL—control condition.

**FIGURE 4:**
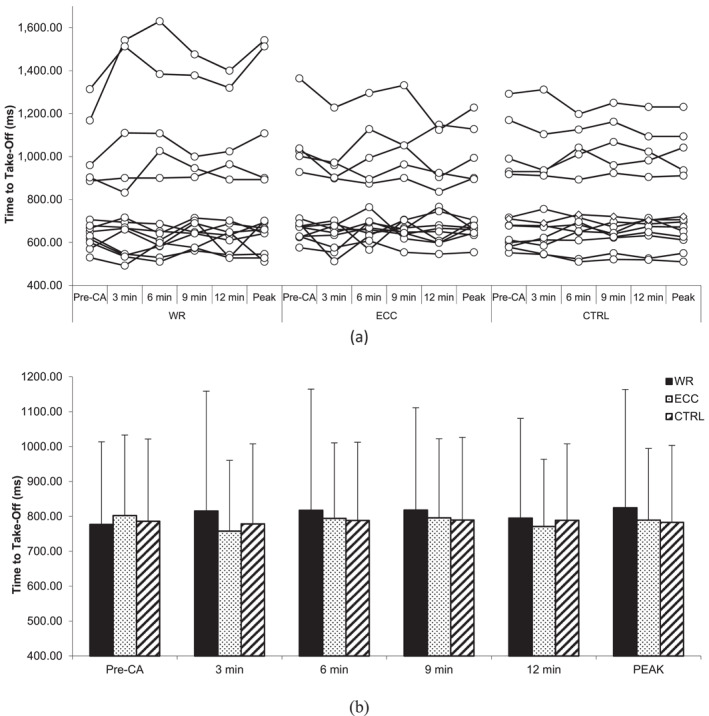
Comparison of pre‐CA and post‐CA countermovement jump time to take‐off. Results are presented as individual responses (A) and mean ± *SD* (B); CA—conditioning activity; PEAK—optimal rest time interval; WR—eccentric‐concentric condition; ECC—eccentric‐only condition; CTRL—control condition.

No statistically significant interactions for CMJ height (*F* = 1.475, *p* = 0.175, and *η* ^2^ = 0.024), relative peak power output (*F* = 0.303, *p* = 0.963, and *η* ^2^ = 0.014), countermovement depth (*F* = 1.159, *p* = 0.331, and *η* ^2^ = 0.023), and time to take‐off (*F* = 0.666, *p* = 0.72, and *η* ^2^ = 0.012) were revealed. Similarly, there was no statistically significant main effect of the condition for CMJ height (*F* = 1.268, *p* = 0.298, and *η* ^2^ = 0.056), relative peak power output (*F* = 0.187, *p* = 0.831, and *η* ^2^ = 0.006), countermovement depth (*F* = 1.966, *p* = 0.16, and *η* ^2^ = 0.077), and time to take‐off (*F* = 0.506, *p* = 0.609, *η* ^2^ = 0.024) nor a main effect of time (*F* = 2.340, *p* = 0.067, and *η* ^2^ = 0.021; *F* = 0.345, *p* = 0.846, and *η* ^2^ = 0.01; *F* = 2.359, *p* = 0.065, and *η* ^2^ = 0.02; *F* = 1.531, *p* = 0.207, and *η* ^2^ = 0.01) (Figures 1, 2, 3, 4).

However, two‐way ANOVA for CMJ height at peak performance indicated a statistically significant interaction (*F* = 4.486, *p* = 0.021, *η* ^2^ = 0.061). CMJ height post‐CA at peak performance during the ECC‐CON condition was higher (39.8 ± 5.0 cm) than pre‐CA (38.1 ± 5.2 cm; *t* (13) = 3.52, *p* = 0.003, *δ* = 1.7 cm [95%CI 0.66–2.74], *Δ*% = 4.9 [95%CI 2%–7.8%]). The CLES indicates that the probability of a randomly selected individual's CMJ height increasing after ECC‐CON CA at peak performance is 83%. On the other hand, there were no statistically significant interactions for CMJ relative peak power output at peak performance (*F* = 1.622, *p* = 0.217, and *η* ^2^ = 0.021) nor the main effect of condition (*F* = 0.012, *p* = 0.988, and *η* ^2^ < 0.001), as well as the main effect of time (*F* = 2.465, *p* = 0.14, and *η* ^2^ = 0.069).

### 4.2 Muscle mechanical properties

No statistically significant interactions for muscle tone (*F* = 1.296, *p* = 0.254, and *η* ^2^ = 0.027) and stiffness (*F* = 0.480, *p* = 0.868, and *η* ^2^ = 0.012) were revealed. Similarly, there was no statistically significant main effect of the condition for muscle tone (*F* = 1.511, *p* = 0.239, and *η* ^2^ = 0.063) and stiffness (*F* = 1.031, *p* = 0.371, *η* ^2^ = 0.036), nor a main effect of time (*F* = 0.601, *p* = 0.663, and *η* ^2^ = 0.004 and *F* = 0.267, *p* = 0.898, and *η* ^2^ = 0.004) (Table 1).

**TABLE 1:** Comparison of pre‐CA and post‐CA muscle mechanical properties of the vastus lateralis muscle.^a^

		Pre‐CA	3 min post‐CA	6 min post‐CA	9 min post‐CA	12 min post‐CA	Peak
Stiffness (N·m^−1^)	ECC‐CON	343 ± 47 (316–370)	346 ± 44 (320–371)	349 ± 44 (324–375)	345 ± 37 (324–366)	346 ± 37 (324–367)	347 ± 3 6 (326–369)
	ECC	349 ± 51 (319–379)	365 ± 64 (328–402)	355 ± 50 (354–383)	352 ± 67 (313–390)	355 ± 66 (317–393)	362 ± 61 (327–398)
	CTRL	345 ± 51 (315–374)	341 ± 55 (310–373)	341 ± 48 (313–368)	344 ± 51 (315–374)	340 ± 47 (313–367)	338 ± 48 (310–366)
Muscle tone (Hz)	ECC‐CON	18.43 ± 2.35 (17.08–19.79)	18.54 ± 2.18 (17.28–19.80)	18.56 ± 2.35 (17.20–19.91)	18.32 ± 1.96 (17.19–19.45)	18.05 ± 1.87 (16.97–19.13)	18.44 ± 1.99 (17.29–19.59)
	ECC	18.31 ± 2.01 (17.15–9.47)	18.74 ± 2.34 (17.39–0.09)	18.85 ± 2.91 (17.17–0.53)	19.07 ± 1.86 (17.41–0.72)	19.00 ± 2.74 (17.42–0.58)	18.97 ± 2.07 (17.78–0.17)
	CTRL	18.38 ± 1.41 (17.56–9.19)	17.62 ± 1.57 (16.71–8.52)	17.84 ± 1.89 (16.75–8.93)	18.14 ± 2.23 (16.85–19.43)	17.68 ± 2.16 (16.44–18.93)	18.07 ± 2.32 (16.73–19.40)

*Note*: Values expressed as mean, standard deviation and 95% confidence interval.

^a^ CA = conditioning activity; ECC‐CON = eccentric‐concentric condition; ECC = eccentric condition; CTRL = control condition. Results are presented as mean ± SD and 95% confidence interval.

Similarly, no statistically significant interactions for muscle tone (*F* = 1.147, *p* = 0.333, and *η* ^2^ = 0.033) and stiffness (*F* = 0.899, *p* = 0.419, and *η* ^2^ = 0.029) at peak performance were found. Moreover, there was no statistically significant main effect of the condition for muscle tone (*F* = 0.669, *p* = 0.521, and *η* ^2^ = 0.024) and stiffness (*F* = 2.348, *p* = 0.116, and *η* ^2^ = 0.064) nor a main effect of time (*F* = 0.356, *p* = 0.561, and *η* ^2^ = 0.003 and *F* = 0.609, *p* = 0.449, and *η* ^2^ = 0.006).

### 4.3 Skin surface temperature

**FIGURE 5:**
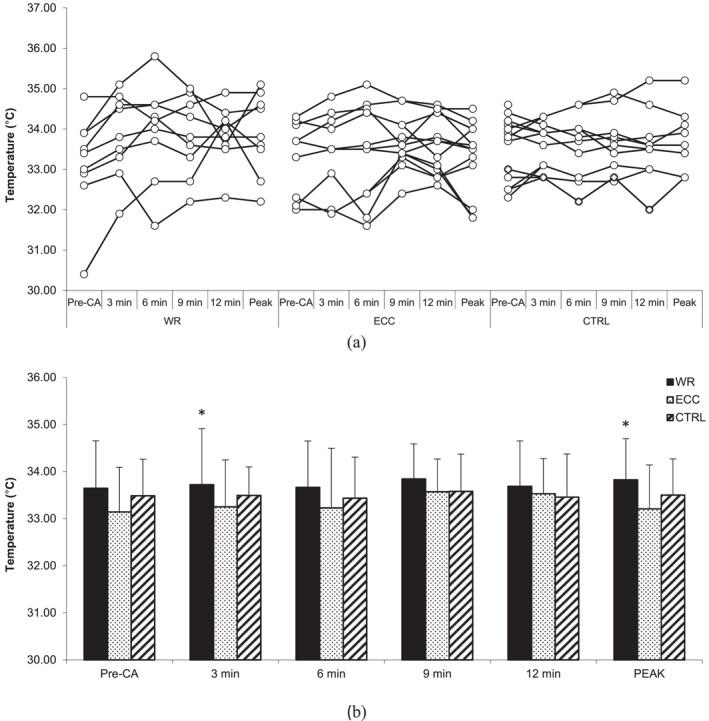
Comparison of pre‐CA and post‐CA front thigh skin surface temperature. Results are presented as individual responses (A) and mean ± *SD* (B); CA—conditioning activity; PEAK—optimal rest time interval; WR—eccentric‐concentric condition; ECC—eccentric‐only condition; CTRL—control condition. * significantly higher than the pre‐CA protocol.

Two‐way ANOVA for skin surface temperature indicated a statistically significant interaction (*F* = 3.922, *p* < 0.001, *η* ^2^ = 0.084). Skin surface temperature at third min after CA during the ECC‐CON condition was higher (33.64 ± 1.01°C) than pre‐CA (32.98 ± 1.24°C; *t* (Douglas et al., 2017) = 5.29, *p* = 0.001, *δ* = 0.66°C [95%CI 0.39–0.93], *Δ* = 2.1% [95%CI 1.2%–3%) (Figure 5). The CLES indicates that the probability of a randomly selected individual's skin surface temperature increasing after at third min after ECC‐CON CA is 92%.

Similarly, a statistically significant interaction for skin surface temperature at peak performance (*F* = 5.304, *p* = 0.023, *η* ^2^ = 0.11) was revealed. Skin surface temperature post‐CA at peak performance during the ECC‐CON condition was higher (33.69 ± 0.96°C) than pre‐CA (32.98 ± 1.24°C; *t* (Douglas et al., 2017) = 3.28, *p* = 0.006, *δ* = 0.71°C [95%CI 0.44–0.98], *Δ*% = 2.2% [95%CI 0.7%–3.7%]). The CLES indicates that the probability of a randomly selected individual's skin surface temperature increasing after ECC‐CON CA at peak performance is 81%.

## 5 DISCUSSION

The main objective of this experiment was to assess the acute effects of performing the ECC and ECC‐CON BS both supramaximal loaded on subsequent CMJ performance. Additionally, the study aimed to investigate changes in the front thigh skin surface temperature and vastus lateralis stiffness and tonus following the mentioned BS conditions. The primary outcome of this research indicates that only the ECC‐CON protocol (2 sets of 2 repetitions at 110% 1RM; distributed by half on the barbell and WR) led to a significant enhancement in CMJ height. However, this effect was noticed only when an individually optimal rest period was used. Furthermore, a significant increase in the skin surface temperature of the front thigh has been observed. In contrast, it was shown that none of the implemented CAs had a statistically significant impact on the mechanical properties of the vastus lateralis muscle.

To the best of the authors' knowledge, this is the first study to date that investigated the acute impact of performing only the eccentric phase of supramaximal loaded BS on the PAPE effect, verified through CMJ with a concomitant assessment of front thigh skin surface temperature and mechanical properties of vastus lateralis. The results of this study are partially in contrast with previous findings analyzing the use of supramaximal loaded CA performed in an eccentric‐only manner on the PAPE effect (Krzysztofik et al., 2020, 2022). Although the same CA parameters as in the study by Krzysztofik et al. (2020) were applied in the protocol of the current study, the only significant CMJ performance enhancement was found after an optimal rest interval post‐CA. In turn, in a study by Krzysztofik et al. (2020), the PAPE effect appeared in a more uniform manner, and a significant improvement in peak power output during bench press throw was noted in the sixth min after 2 sets of 2 repetitions of bench press at 110% 1RM. It seems that the differing outcomes could be attributed to differences between upper and lower limb exercises. While lower‐body exercises allow for the application of higher absolute external load and pose greater physiological demands on the athlete (Andrade et al., 2022), they also represent a significantly larger muscle area compared to the upper body (Janssen et al., 2000). Therefore, it is plausible that inducing a significant PAPE effect in the lower limb protocol might require higher intensity or/and volume compared to upper limb exercises. This could be supported by the observed increase in CMJ height following ECC‐CON but not after the ECC condition in this study. It has been shown that eccentric action alters the PAPE effect compared to stretch‐shortening cycle CA (Stastny et al., 2024) in particular muscle groups with possibly no effect on jump height, which was observed also in our results. However, the balance between potentiation and fatigue caused by the CA (Tillin et al., 2009) includes also the similarity in resembling the movement pattern of used muscle groups, where the contrast of highly loaded eccentric action followed by unloaded concentric pattern might have a higher CA effect than manipulation with the load and volume.

This study is probably the second of its kind to assess the efficacy of using WR in PAPE protocols (Tseng et al., 2021). The study by Tseng et al. (2021), in contrast to the current investigation, did not show a significant impact of BS (3 sets of 4 repetitions with a 3‐min rest interval between sets) at 105% 1RM during the eccentric phase and 80% 1RM during the concentric phase (a 25% 1RM was unloaded by WR) on CMJ performed 10 min later. It should be noted, however, that the research procedures significantly differed. Firstly, this study employed a threefold lower CA volume, specifically 2 sets of 2 repetitions with a 3‐min rest interval between sets. Additionally, the magnitude and distribution of the external load on the barbell and WR were substantially different. In this study, the eccentric phase of the BS was performed at 110% 1RM, while the concentric phase was at 55% 1RM. This suggests that the effectiveness of utilizing supramaximal loads in lower limb PAPE protocols might be more dependent on intensity rather than volume (Merrigan et al., 2022). Thus, in the case of lower limbs PAPE protocols, it may be necessary to apply higher external loads compared to upper limb CAs. On the other hand, the CA volume used by Tseng et al. (2021) study was significantly larger than in the current experiment, thus pointing to the need for studies that would directly compare the different supramaximal CA volumes in the upper and lower body PAPE protocols.

Furthermore, it is worth noting that in the study by Tseng et al. (2021), the PAPE effect was assessed solely at a one‐time point after CA, whereas in the current investigation, measurements were taken at the third, sixth, ninth, and 12^th^ minute after CA. When the entire group was analyzed at each time point, this study also did not reveal a significant impact of the applied CA on CMJ performance. However, when the optimal rest interval was evaluated, a significant ECC‐CON effect on CMJ height became evident. This points to the need for the high individualization of employed PAPE protocols, particularly regarding the post‐CA rest interval, as reported in previous studies (Chiu et al., 2003; Krzysztofik, Wilk, Pisz, Kolinger, Tsoukos, et al., 2023). Another distinction worth mentioning is the differing muscle strength levels of the participants in this study compared to Tseng et al. (2021). The mean relative muscle strength level in BS for the participants of Tseng et al. (2021) was 1.90 kg/kg of body mass, whereas, in this study, participants exhibited a slightly higher level of 2.10 kg/kg of body mass. The literature clearly indicates that the level of an individual's muscle strength plays a crucial role in the occurrence and magnitude of the PAPE effect, which can be explained by greater fatigue resistance in stronger individuals compared to their weaker counterparts (Chiu et al., 2003; Seitz et al., 2016). Therefore, it might seem that the higher muscle strength level of the participants in this study influenced the occurrence of the PAPE effect following ECC‐CON. Moreover, it should be highlighted that the protocol used in this study was effective in eliciting the PAPE effect in nine of the participants, while four of them experienced no significant PAPE effect, and one responded negatively. Hence, the ECC‐CON protocol used in this study was more likely to elicit the PAPE effect than ECC‐only. However, more research is needed comparing supramaximal loads with typical PAPE protocols in groups of highly trained and strong athletes.

Additionally, this study observed a significant rise in the front thigh skin surface temperature following ECC‐CON. This temperature increase might be associated with the execution of the concentric phase during the ECC‐CON condition, a phase that was absent in the ECC condition. The energy needs of eccentric contractions are notably lower in comparison to concentric contractions (Douglas et al., 2017). This is attributed to the prominent engagement of passive components during eccentric contractions, as highlighted by Hessel et al. (2017). Therefore, it is reasonable to suggest that this leads to a reduction in thermal energy emission. The observed increase in skin surface temperature, despite a small number of repetitions in the given CA (just 4 repetitions), is contradictory to the results of previous research that have examined the impact of resistance exercise on skin surface temperature changes (Weigert et al., 2018). Nonetheless, differences resulted from the significantly larger muscle area engagement during BS compared to elbow flexion exercise, as in the study by Weigert et al. (2018), cannot be excluded. In the latter study, a decrease in skin surface temperature was observed after performing 10 repetitions of the mentioned exercise. Hence, the hypothetically higher heat‐generating potential during muscle work in the lower limbs compared to the upper limbs might explain the noted increase in front thigh skin surface temperature (González‐Alonso et al., 2000). Nevertheless, Formenti et al. (2016) showed that the speed at which exercise phases are performed has an impact on changes in skin surface temperature. More specifically, a more rapid decline in temperature was found when the eccentric and concentric phases were executed at a faster speed (1 vs. 5s). Hence, the implementation of a 3‐s eccentric phase in the protocols employed in this study (as opposed to the 1.5‐s duration in the study conducted by Weigert et al. (2018)) could potentially be responsible for the observed rise in skin surface temperature. However, it is crucial to remember that skin surface temperature is a method that mainly offers insights into the thermoregulatory response. It indicates changes in the superficial tissue due to temperature fluctuations in the active muscle regions but does not directly measure the internal muscle temperature.

Another aspect that has not been extensively analyzed in studies investigating the PAPE effect is the impact of the CA on the muscle's mechanical properties (Krzysztofik, Spieszny, et al., 2023; Krzysztofik, Wilk, Pisz, Kolinger, Bichowska, et al., 2023; Krzysztofik, Wilk, Pisz, Kolinger, Tsoukos, et al., 2023). Considering that potential mechanisms underlying the PAPE effect include an increase in intramuscular fluid volume (Blazevich et al., 2019) and that a positive relationship exists between muscle mechanical properties and intramuscular fluid pressure (Korhonen et al., 2005), those assessments might somehow explain changes in athletic performance after applied CA. The influence of intramuscular fluid pressure on muscle performance is noteworthy since it may temporarily alter muscle fibers architecture and therefore affect the magnitude of the force exerted (Sejersted et al., 1984). The current study showed no significant changes in vastus lateralis stiffness and muscle tone, despite observing a significant improvement in CMJ height following the ECC‐CON condition. Although the fact that an increase in intramuscular fluid has been suggested as a potential mechanism explaining the PAPE effect, recent research indicates that it is more closely associated with fatigue rather than improvements in physical performance due to impaired clearance of metabolic byproducts. For instance, Klich et al. (2020) reported an acute rise in vastus lateralis stiffness following all‐out 200 and 4000 m track cycling with a more pronounced increase after the shorter sprint. Moreover, Trybulski et al. (2022) noted a trend toward increased muscle stiffness in the triceps brachii long head along with a simultaneous reduction in barbell velocity during the bench press exercise. Following this line of thought, the absence of significant changes in stiffness and muscle tone may indicate a lack of significant disturbances in intramuscular fluid pressure and, consequently, fatigue.

Summarizing, the ECC‐CON supramaximal lower limb PAPE protocol can be effectively used among males representing very high levels of lower limb muscle strength (>2 × body mass). Additionally, considering the large variability in PAPE responses observed across athletes, the study's findings suggest that it is crucial to individually assess the effectiveness of such a protocol along with assessments of the optimal rest interval after a CA in order to establish whether athletes respond positively and could experience the PAPE effect following supramaximal loaded BS.

## CONFLICT OF INTEREST STATEMENT

The authors declare no conflicts of interest.

## PATIENT CONSENT STATEMENT

Informed consent was obtained from all subjects involved in the study.

## PERMISSION TO REPRODUCE MATERIAL FROM OTHER SOURCES

Not applicable.

## CLINICAL TRIAL REGISTRATION

Not applicable.

## Data Availability

Data is available on request from the corresponding author.
